# 1-Dibromo­methyl-4-meth­oxy-2-nitro­benzene

**DOI:** 10.1107/S1600536809031833

**Published:** 2009-08-19

**Authors:** Hoong-Kun Fun, Jia Hao Goh, B. Chandrakantha, Arun M. Isloor

**Affiliations:** a X-ray Crystallography Unit, School of Physics, Universiti Sains Malaysia, 11800 USM, Penang, Malaysia; b Syngene International Ltd, Biocon Park, Plot Nos. 2 & 3, Bommasandra 4th Phase, Jigani Link Road, Bangalore 560 100, India; c Department of Chemistry, National Institute of Technology-Karnataka, Surathkal, Mangalore 575 025, India

## Abstract

The asymmetric unit of the title compound, C_8_H_7_Br_2_NO_3_, comprises two crystallographically independent mol­ecules (*A* and *B*). The nitro groups are twisted from the attached benzene rings, making dihedral angles of 39.26 (9) and 35.90 (9)° in mol­ecules *A* and *B*, respectively. In each mol­ecule, the dibromo­methyl group is orientated in such a way that the two Br atoms are tilted away from the benzene ring. An inter­esting features of the crystal structure is the two short Br⋯Br inter­actions which, together with inter­molecular C—H⋯O hydrogen bonds, link the mol­ecules into an extended three-dimensional network. The crystal structure is further stabilized by weak C—H⋯π inter­actions.

## Related literature

For general background to and applications of brominated organic compounds, see Augustine *et al.* (2007 ▶); Derdau *et al.* (2003 ▶); Khatuya (2001 ▶); Tyeklar *et al.* (1993 ▶). For related structures, see: Fun, Chantrapromma, Maity *et al.* (2009 ▶); Fun, Chantrapromma, Sujith *et al.* (2009 ▶); Yeap *et al.* (2008 ▶). For the stability of the temperature controller used in the data collection, see: Cosier & Glazer (1986 ▶).
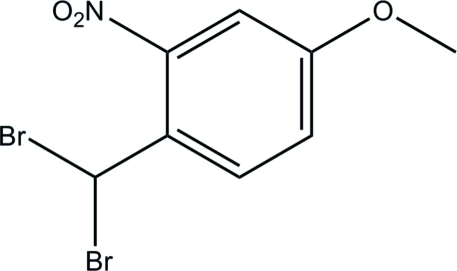


## Experimental

### 

#### Crystal data

C_8_H_7_Br_2_NO_3_
*M* *_r_* = 324.97
Triclinic, 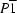

*a* = 7.9591 (1) Å
*b* = 11.1949 (2) Å
*c* = 12.2509 (2) Å
α = 106.285 (1)°
β = 99.691 (1)°
γ = 102.401 (1)°
*V* = 992.45 (3) Å^3^
*Z* = 4
Mo *K*α radiation
μ = 8.15 mm^−1^
*T* = 100 K
0.28 × 0.25 × 0.19 mm

#### Data collection

Bruker SMART APEXII CCD area-detector diffractometer
Absorption correction: multi-scan (**SADABS**; Bruker, 2005 ▶) *T*_min_ = 0.210, *T*_max_ = 0.311 (expected range = 0.147–0.218)
32659 measured reflections
8800 independent reflections
7332 reflections with *I* > 2σ(*I*)
*R*_int_ = 0.027

#### Refinement

*R*[*F* ^2^ > 2σ(*F* ^2^)] = 0.026
*wR*(*F* ^2^) = 0.066
*S* = 1.01
8800 reflections
261 parameters
H atoms treated by a mixture of independent and constrained refinement
Δρ_max_ = 0.78 e Å^−3^
Δρ_min_ = −0.47 e Å^−3^

### 

Data collection: *APEX2* (Bruker, 2005 ▶); cell refinement: *SAINT* (Bruker, 2005 ▶); data reduction: *SAINT*; program(s) used to solve structure: *SHELXTL* (Sheldrick, 2008 ▶); program(s) used to refine structure: *SHELXTL*; molecular graphics: *SHELXTL*; software used to prepare material for publication: *SHELXTL* and *PLATON* (Spek, 2009 ▶).

## Supplementary Material

Crystal structure: contains datablocks global, I. DOI: 10.1107/S1600536809031833/wn2343sup1.cif

Structure factors: contains datablocks I. DOI: 10.1107/S1600536809031833/wn2343Isup2.hkl

Additional supplementary materials:  crystallographic information; 3D view; checkCIF report

## Figures and Tables

**Table 1:** Selected interatomic distances (Å)

Br1*A*⋯Br2*B*^i^	3.5915 (3)
Br2*A*⋯Br1*B*^ii^	3.6279 (2)

Symmetry codes: (i) 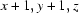; (ii) 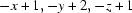.

**Table 2:** Hydrogen-bond geometry (Å, °)

*D*—H⋯*A*	*D*—H	H⋯*A*	*D*⋯*A*	*D*—H⋯*A*
C7*A*—H7*A*⋯O2*B*	0.95 (2)	2.47 (2)	3.134 (2)	126.8 (17)
C8*B*—H8*BA*⋯O1*A*^iii^	0.96	2.52	3.370 (2)	148
C8*A*—H8*AA*⋯*Cg*2^iv^	0.96	2.95	3.839 (2)	155

Symmetry codes: (iii) 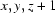; (iv) 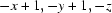. *Cg*2 is the centroid of the C1*B*–C6*B* benzene ring.

**Fig. 1.**
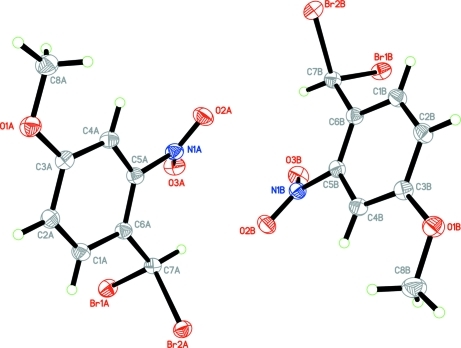
The molecular structure of the asymmetric unit of the title compound, showing 50% probability displacement ellipsoids and the atom-numbering scheme. Hydrogen atoms are shown as spheres of arbitrary radius.

**Fig. 2.**
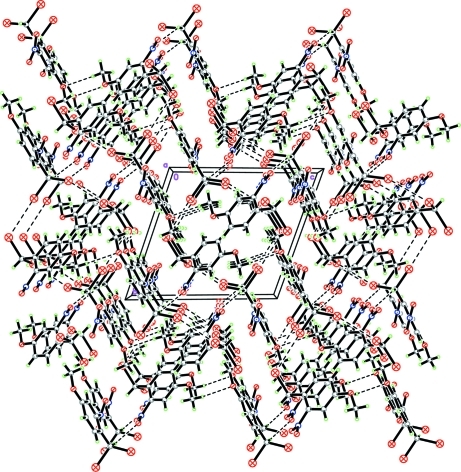
Three-dimensional extended network, viewed along the a axis. Intermolecular interactions are shown as dashed lines.

## supplementary crystallographic information

### Comment

Brominated organic compounds are important synthetic intermediates and products in organic chemistry (Augustine *et al.*, 2007). They are found in C-C coupling reactions, as precursors to organometallic species and in nucleophilic substitutions (Tyeklar *et al.*, 1993). They are also used for the synthesis of useful pharmaceutical materials and agrochemicals (Derdau *et al.*, 2003). However the use of molecular bromine as an electrophilic brominating reagent has several drawbacks arising from its toxic and corrosive nature and its high reactivity (Tyeklar *et al.*, 1993). Alternative brominating reagents such as N-bromosuccinimide make for easier handling and result in improved selectivity (Khatuya, 2001).

In the asymmetric unit of the title compound, there are two crystallographically independent molecules, designated *A* and *B* (Fig. 1). In each molecule, the nitro group is twisted from the mean plane of the C1-C6 benzene ring, as shown by the dihedral angle formed between the mean plane through C5/N1/O2/O3 and the C1-C6 benzene ring of 39.26 (9)° in molecule *A*; the comparable angle is 35.90 (9)° for molecule *B*. Meanwhile, the dibromomethyl group is orientated in such a way that the two Br atoms are tilted away from the benzene ring. The bond lengths and angles are comparable to those found in related structures (Fun, Chantrapromma, Maity *et al.*, 2009; Fun, Chantrapromma, Sujith *et al.*, 2009; Yeap *et al.*, 2008).

In the crystal structure (Fig. 2), the interesting features are the Br1A···Br2B and Br2A···Br1B short interactions (Table 1). Together with intermolecular C7A—H7A···O2B and C8B—H8BA···O1A hydrogen bonds (Table 2), they link the molecules into a three-dimensional extended network. The crystal structure is further stabilized by weak C8A—H8AA···*Cg*2 interactions (Table 2).

### Experimental

Benzoyl peroxide (0.20 g, 10 %) and N-bromosuccinimide (6.38 g, 0.0358 mol) were added in portions to a solution of 4-methyl-2-nitroanisole (2.00 g, 0.0119 mol) in CCl_4_ (20 ml). The reaction mixture was heated at 85 °C under a nitrogen atmosphere for 12 h. The reaction mass was cooled and filtered. The filtrate was concentrated to produce a crude product. The latter was recrystallized with hexane to afford the title compound as a colourless crystalline solid. The yield was 3.50 g, 92 %. *M.p.* 370–373 K.

### Refinement

The H-atoms bound to C7A and C7B were located from the difference Fourier map and allowed to refine freely. The other H-atoms were placed in calculated positions, with C—H = 0.93 Å, *U*_iso_(H) = 1.2*U*_eq_(C) for aromatic, and C—H = 0.96 Å, *U*_iso_(H) = 1.5*U*_eq_(C) for methyl group; these aromatic and methyl group H atoms were refined as riding on their parent atoms. A rotating group model was used for the methyl group.

### Crystal data

**Table:**

C_8_H_7_Br_2_NO_3_	*Z* = 4
*M**_r_* = 324.97	*F*(000) = 624
Triclinic, *P*1	*D*_x_ = 2.175 Mg m^−^^3^
Hall symbol: -P 1	Mo *K*α radiation, λ = 0.71073 Å
*a* = 7.9591 (1) Å	Cell parameters from 9885 reflections
*b* = 11.1949 (2) Å	θ = 2.2–35.1°
*c* = 12.2509 (2) Å	µ = 8.15 mm^−^^1^
α = 106.285 (1)°	*T* = 100 K
β = 99.691 (1)°	Block, colourless
γ = 102.401 (1)°	0.28 × 0.25 × 0.19 mm
*V* = 992.45 (3) Å^3^	

### Data collection

**Table:**

Bruker SMART APEXII CCD area-detector diffractometer	8800 independent reflections
Radiation source: fine-focus sealed tube	7332 reflections with *I* > 2σ(*I*)
graphite	*R*_int_ = 0.027
φ and ω scans	θ_max_ = 35.3°, θ_min_ = 2.0°
Absorption correction: multi-scan (*SADABS*; Bruker, 2005)	*h* = −12→12
*T*_min_ = 0.210, *T*_max_ = 0.311	*k* = −17→18
32659 measured reflections	*l* = −19→19

### Refinement

**Table:**

Refinement on *F*^2^	Primary atom site location: structure-invariant direct methods
Least-squares matrix: full	Secondary atom site location: difference Fourier map
*R*[*F*^2^ > 2σ(*F*^2^)] = 0.026	Hydrogen site location: inferred from neighbouring sites
*wR*(*F*^2^) = 0.066	H atoms treated by a mixture of independent and constrained refinement
*S* = 1.01	*w* = 1/[σ^2^(*F*_o_^2^) + (0.0361*P*)^2^ + 0.2346*P*] where *P* = (*F*_o_^2^ + 2*F*_c_^2^)/3
8800 reflections	(Δ/σ)_max_ = 0.004
261 parameters	Δρ_max_ = 0.78 e Å^−^^3^
0 restraints	Δρ_min_ = −0.47 e Å^−^^3^

### Special details

**Table:**

Experimental. The crystal was placed in the cold stream of an Oxford Cyrosystems Cobra open-flow nitrogen cryostat (Cosier & Glazer, 1986) operating at 100.0 (1)K.
Geometry. All esds (except the esd in the dihedral angle between two l.s. planes) are estimated using the full covariance matrix. The cell esds are taken into account individually in the estimation of esds in distances, angles and torsion angles; correlations between esds in cell parameters are only used when they are defined by crystal symmetry. An approximate (isotropic) treatment of cell esds is used for estimating esds involving l.s. planes.
Refinement. Refinement of F^2^ against ALL reflections. The weighted R-factor wR and goodness of fit S are based on F^2^, conventional R-factors R are based on F, with F set to zero for negative F^2^. The threshold expression of F^2^ > 2sigma(F^2^) is used only for calculating R-factors(gt) etc. and is not relevant to the choice of reflections for refinement. R-factors based on F^2^ are statistically about twice as large as those based on F, and R- factors based on ALL data will be even larger.

### Fractional atomic coordinates and isotropic or equivalent isotropic displacement parameters (Å^2^)

**Table:**

	*x*	*y*	*z*	*U*_iso_*/*U*_eq_	
Br1A	0.79537 (2)	1.230776 (16)	0.197580 (14)	0.02458 (4)	
Br2A	0.90786 (2)	1.101536 (16)	0.387687 (13)	0.02180 (4)	
O1A	0.76010 (16)	0.62902 (12)	−0.10724 (10)	0.0229 (2)	
O2A	0.30267 (16)	0.76549 (13)	0.10327 (11)	0.0265 (2)	
O3A	0.37293 (16)	0.96938 (13)	0.11963 (11)	0.0257 (2)	
N1A	0.40781 (17)	0.86506 (14)	0.10586 (11)	0.0208 (2)	
C1A	0.8937 (2)	0.94163 (16)	0.11837 (13)	0.0209 (3)	
H1AA	0.9990	1.0061	0.1578	0.025*	
C2A	0.8985 (2)	0.83283 (16)	0.03310 (13)	0.0216 (3)	
H2AA	1.0052	0.8260	0.0142	0.026*	
C3A	0.7427 (2)	0.73241 (15)	−0.02523 (13)	0.0195 (3)	
C4A	0.58321 (19)	0.74374 (15)	0.00283 (13)	0.0188 (2)	
H4AA	0.4792	0.6770	−0.0335	0.023*	
C5A	0.58299 (19)	0.85699 (15)	0.08631 (13)	0.0185 (2)	
C6A	0.73536 (19)	0.95856 (15)	0.14790 (13)	0.0184 (2)	
C7A	0.7370 (2)	1.07568 (15)	0.24351 (13)	0.0196 (3)	
C8A	0.6054 (2)	0.52136 (17)	−0.16335 (15)	0.0256 (3)	
H8AA	0.6344	0.4546	−0.2193	0.038*	
H8AB	0.5141	0.5491	−0.2029	0.038*	
H8AC	0.5640	0.4883	−0.1055	0.038*	
Br1B	−0.20637 (2)	0.726206 (17)	0.325018 (14)	0.02462 (4)	
Br2B	−0.10244 (2)	0.523519 (16)	0.125556 (13)	0.02342 (4)	
O1B	0.50849 (16)	0.63292 (12)	0.60857 (11)	0.0243 (2)	
O2B	0.49443 (16)	0.92884 (12)	0.36858 (11)	0.0252 (2)	
O3B	0.22098 (17)	0.93622 (12)	0.34859 (12)	0.0261 (2)	
N1B	0.33898 (17)	0.88484 (13)	0.36914 (11)	0.0197 (2)	
C1B	0.1045 (2)	0.56681 (15)	0.38962 (13)	0.0206 (3)	
H1BA	−0.0019	0.5017	0.3575	0.025*	
C2B	0.2309 (2)	0.55536 (16)	0.47501 (14)	0.0215 (3)	
H2BA	0.2081	0.4840	0.5005	0.026*	
C3B	0.3935 (2)	0.65079 (16)	0.52360 (13)	0.0200 (3)	
C4B	0.4281 (2)	0.75603 (15)	0.48395 (13)	0.0199 (3)	
H4BA	0.5370	0.8186	0.5131	0.024*	
C5B	0.29538 (19)	0.76575 (15)	0.39929 (13)	0.0180 (2)	
C6B	0.13101 (19)	0.67357 (15)	0.34948 (13)	0.0185 (2)	
C7B	−0.0114 (2)	0.68337 (16)	0.25833 (13)	0.0203 (3)	
C8B	0.6731 (2)	0.73163 (19)	0.66117 (16)	0.0289 (3)	
H8BA	0.7436	0.7088	0.7196	0.043*	
H8BB	0.6504	0.8126	0.6971	0.043*	
H8BC	0.7359	0.7397	0.6021	0.043*	
H7A	0.627 (3)	1.0704 (19)	0.2645 (17)	0.013 (4)*	
H7B	0.022 (3)	0.747 (2)	0.226 (2)	0.028 (6)*	

### Atomic displacement parameters (Å^2^)

**Table:**

	*U*^11^	*U*^22^	*U*^33^	*U*^12^	*U*^13^	*U*^23^
Br1A	0.03013 (8)	0.01907 (7)	0.02323 (7)	0.00585 (6)	0.00401 (6)	0.00714 (6)
Br2A	0.01964 (7)	0.02534 (8)	0.01851 (6)	0.00619 (5)	0.00280 (5)	0.00539 (5)
O1A	0.0221 (5)	0.0217 (5)	0.0231 (5)	0.0078 (4)	0.0056 (4)	0.0033 (4)
O2A	0.0185 (5)	0.0262 (6)	0.0315 (6)	0.0021 (4)	0.0067 (4)	0.0068 (5)
O3A	0.0211 (5)	0.0255 (6)	0.0293 (6)	0.0111 (4)	0.0033 (4)	0.0052 (5)
N1A	0.0171 (5)	0.0232 (6)	0.0198 (5)	0.0060 (5)	0.0029 (4)	0.0043 (5)
C1A	0.0167 (6)	0.0229 (7)	0.0215 (6)	0.0049 (5)	0.0037 (5)	0.0060 (5)
C2A	0.0180 (6)	0.0255 (7)	0.0210 (6)	0.0071 (5)	0.0051 (5)	0.0062 (5)
C3A	0.0206 (6)	0.0198 (7)	0.0185 (6)	0.0076 (5)	0.0039 (5)	0.0061 (5)
C4A	0.0173 (6)	0.0179 (6)	0.0196 (6)	0.0050 (5)	0.0020 (5)	0.0051 (5)
C5A	0.0158 (6)	0.0200 (7)	0.0199 (6)	0.0062 (5)	0.0037 (5)	0.0063 (5)
C6A	0.0173 (6)	0.0189 (6)	0.0186 (6)	0.0051 (5)	0.0036 (5)	0.0060 (5)
C7A	0.0191 (6)	0.0186 (6)	0.0189 (6)	0.0034 (5)	0.0034 (5)	0.0046 (5)
C8A	0.0278 (8)	0.0205 (7)	0.0260 (7)	0.0066 (6)	0.0064 (6)	0.0042 (6)
Br1B	0.01982 (7)	0.02733 (8)	0.02389 (7)	0.00989 (6)	0.00277 (5)	0.00308 (6)
Br2B	0.02590 (7)	0.02424 (8)	0.01841 (6)	0.00830 (6)	0.00365 (5)	0.00441 (5)
O1B	0.0208 (5)	0.0264 (6)	0.0264 (5)	0.0062 (4)	0.0013 (4)	0.0125 (5)
O2B	0.0205 (5)	0.0262 (6)	0.0282 (6)	0.0014 (4)	0.0061 (4)	0.0118 (5)
O3B	0.0262 (6)	0.0224 (6)	0.0327 (6)	0.0100 (5)	0.0059 (5)	0.0122 (5)
N1B	0.0209 (6)	0.0174 (6)	0.0202 (5)	0.0043 (5)	0.0045 (4)	0.0065 (4)
C1B	0.0197 (6)	0.0189 (7)	0.0225 (6)	0.0038 (5)	0.0042 (5)	0.0076 (5)
C2B	0.0211 (6)	0.0197 (7)	0.0251 (7)	0.0055 (5)	0.0058 (5)	0.0094 (5)
C3B	0.0187 (6)	0.0211 (7)	0.0216 (6)	0.0073 (5)	0.0049 (5)	0.0077 (5)
C4B	0.0181 (6)	0.0200 (7)	0.0211 (6)	0.0050 (5)	0.0040 (5)	0.0065 (5)
C5B	0.0191 (6)	0.0165 (6)	0.0196 (6)	0.0055 (5)	0.0057 (5)	0.0066 (5)
C6B	0.0170 (6)	0.0190 (6)	0.0192 (6)	0.0051 (5)	0.0042 (5)	0.0058 (5)
C7B	0.0193 (6)	0.0199 (7)	0.0208 (6)	0.0048 (5)	0.0039 (5)	0.0065 (5)
C8B	0.0228 (7)	0.0303 (9)	0.0301 (8)	0.0047 (6)	−0.0012 (6)	0.0112 (7)

### Geometric parameters (Å, °)

**Table:**

Br1A—C7A	1.9587 (15)	Br1B—C7B	1.9576 (16)
Br2A—C7A	1.9462 (15)	Br2B—C7B	1.9460 (16)
O1A—C3A	1.3535 (19)	O1B—C3B	1.3547 (19)
O1A—C8A	1.433 (2)	O1B—C8B	1.431 (2)
O2A—N1A	1.2306 (18)	O2B—N1B	1.2314 (17)
O3A—N1A	1.2305 (19)	O3B—N1B	1.2288 (18)
N1A—C5A	1.4710 (19)	N1B—C5B	1.4680 (19)
C1A—C2A	1.377 (2)	C1B—C2B	1.378 (2)
C1A—C6A	1.405 (2)	C1B—C6B	1.404 (2)
C1A—H1AA	0.9300	C1B—H1BA	0.9300
C2A—C3A	1.402 (2)	C2B—C3B	1.401 (2)
C2A—H2AA	0.9300	C2B—H2BA	0.9300
C3A—C4A	1.392 (2)	C3B—C4B	1.388 (2)
C4A—C5A	1.388 (2)	C4B—C5B	1.395 (2)
C4A—H4AA	0.9300	C4B—H4BA	0.9300
C5A—C6A	1.397 (2)	C5B—C6B	1.395 (2)
C6A—C7A	1.489 (2)	C6B—C7B	1.497 (2)
C7A—H7A	0.948 (19)	C7B—H7B	0.92 (2)
C8A—H8AA	0.9600	C8B—H8BA	0.9600
C8A—H8AB	0.9600	C8B—H8BB	0.9600
C8A—H8AC	0.9600	C8B—H8BC	0.9600
			
Br1A···Br2B^i^	3.5915 (3)	Br2A···Br1B^ii^	3.6279 (2)
			
C3A—O1A—C8A	117.29 (13)	C3B—O1B—C8B	116.75 (13)
O3A—N1A—O2A	123.97 (14)	O3B—N1B—O2B	123.87 (14)
O3A—N1A—C5A	118.23 (13)	O3B—N1B—C5B	118.83 (12)
O2A—N1A—C5A	117.75 (14)	O2B—N1B—C5B	117.28 (13)
C2A—C1A—C6A	122.27 (14)	C2B—C1B—C6B	122.16 (14)
C2A—C1A—H1AA	118.9	C2B—C1B—H1BA	118.9
C6A—C1A—H1AA	118.9	C6B—C1B—H1BA	118.9
C1A—C2A—C3A	120.06 (14)	C1B—C2B—C3B	120.25 (14)
C1A—C2A—H2AA	120.0	C1B—C2B—H2BA	119.9
C3A—C2A—H2AA	120.0	C3B—C2B—H2BA	119.9
O1A—C3A—C4A	124.35 (14)	O1B—C3B—C4B	124.10 (14)
O1A—C3A—C2A	115.97 (13)	O1B—C3B—C2B	116.25 (14)
C4A—C3A—C2A	119.68 (14)	C4B—C3B—C2B	119.65 (14)
C5A—C4A—C3A	118.44 (14)	C3B—C4B—C5B	118.43 (14)
C5A—C4A—H4AA	120.8	C3B—C4B—H4BA	120.8
C3A—C4A—H4AA	120.8	C5B—C4B—H4BA	120.8
C4A—C5A—C6A	123.78 (14)	C4B—C5B—C6B	123.70 (14)
C4A—C5A—N1A	115.23 (13)	C4B—C5B—N1B	114.52 (13)
C6A—C5A—N1A	120.98 (14)	C6B—C5B—N1B	121.73 (13)
C5A—C6A—C1A	115.70 (14)	C5B—C6B—C1B	115.76 (14)
C5A—C6A—C7A	123.71 (13)	C5B—C6B—C7B	123.88 (14)
C1A—C6A—C7A	120.52 (13)	C1B—C6B—C7B	120.36 (13)
C6A—C7A—Br2A	111.59 (11)	C6B—C7B—Br2B	111.47 (11)
C6A—C7A—Br1A	110.77 (10)	C6B—C7B—Br1B	110.83 (10)
Br2A—C7A—Br1A	108.66 (7)	Br2B—C7B—Br1B	109.65 (7)
C6A—C7A—H7A	113.2 (12)	C6B—C7B—H7B	115.7 (15)
Br2A—C7A—H7A	104.8 (12)	Br2B—C7B—H7B	105.1 (15)
Br1A—C7A—H7A	107.5 (12)	Br1B—C7B—H7B	103.7 (15)
O1A—C8A—H8AA	109.5	O1B—C8B—H8BA	109.5
O1A—C8A—H8AB	109.5	O1B—C8B—H8BB	109.5
H8AA—C8A—H8AB	109.5	H8BA—C8B—H8BB	109.5
O1A—C8A—H8AC	109.5	O1B—C8B—H8BC	109.5
H8AA—C8A—H8AC	109.5	H8BA—C8B—H8BC	109.5
H8AB—C8A—H8AC	109.5	H8BB—C8B—H8BC	109.5
			
C6A—C1A—C2A—C3A	−1.9 (2)	C6B—C1B—C2B—C3B	−1.1 (2)
C8A—O1A—C3A—C4A	−3.9 (2)	C8B—O1B—C3B—C4B	1.5 (2)
C8A—O1A—C3A—C2A	176.29 (14)	C8B—O1B—C3B—C2B	−178.30 (15)
C1A—C2A—C3A—O1A	−179.68 (14)	C1B—C2B—C3B—O1B	178.77 (15)
C1A—C2A—C3A—C4A	0.5 (2)	C1B—C2B—C3B—C4B	−1.1 (2)
O1A—C3A—C4A—C5A	−178.01 (14)	O1B—C3B—C4B—C5B	−177.36 (15)
C2A—C3A—C4A—C5A	1.8 (2)	C2B—C3B—C4B—C5B	2.4 (2)
C3A—C4A—C5A—C6A	−2.9 (2)	C3B—C4B—C5B—C6B	−1.9 (2)
C3A—C4A—C5A—N1A	176.35 (13)	C3B—C4B—C5B—N1B	175.71 (14)
O3A—N1A—C5A—C4A	−139.68 (14)	O3B—N1B—C5B—C4B	−142.98 (15)
O2A—N1A—C5A—C4A	37.79 (19)	O2B—N1B—C5B—C4B	35.46 (19)
O3A—N1A—C5A—C6A	39.6 (2)	O3B—N1B—C5B—C6B	34.7 (2)
O2A—N1A—C5A—C6A	−142.94 (15)	O2B—N1B—C5B—C6B	−146.90 (14)
C4A—C5A—C6A—C1A	1.6 (2)	C4B—C5B—C6B—C1B	−0.1 (2)
N1A—C5A—C6A—C1A	−177.63 (13)	N1B—C5B—C6B—C1B	−177.56 (14)
C4A—C5A—C6A—C7A	−175.35 (14)	C4B—C5B—C6B—C7B	−179.76 (15)
N1A—C5A—C6A—C7A	5.5 (2)	N1B—C5B—C6B—C7B	2.8 (2)
C2A—C1A—C6A—C5A	0.9 (2)	C2B—C1B—C6B—C5B	1.6 (2)
C2A—C1A—C6A—C7A	177.90 (14)	C2B—C1B—C6B—C7B	−178.73 (15)
C5A—C6A—C7A—Br2A	124.47 (14)	C5B—C6B—C7B—Br2B	130.42 (13)
C1A—C6A—C7A—Br2A	−52.30 (17)	C1B—C6B—C7B—Br2B	−49.18 (17)
C5A—C6A—C7A—Br1A	−114.33 (14)	C5B—C6B—C7B—Br1B	−107.15 (14)
C1A—C6A—C7A—Br1A	68.89 (16)	C1B—C6B—C7B—Br1B	73.24 (17)

Symmetry codes: (i) *x*+1, *y*+1, *z*; (ii) −*x*+1, −*y*+2, −*z*+1.

### Hydrogen-bond geometry (Å, °)

**Table:**

*D*—H···*A*	*D*—H	H···*A*	*D*···*A*	*D*—H···*A*
C7A—H7A···O2B	0.95 (2)	2.47 (2)	3.134 (2)	126.8 (17)
C8B—H8BA···O1A^iii^	0.96	2.52	3.370 (2)	148
C8A—H8AA···Cg2^iv^	0.96	2.95	3.839 (2)	155

Symmetry codes: (iii) *x*, *y*, *z*+1; (iv) −*x*+1, −*y*+1, −*z*.
